# The Evolution of Soundscape Appraisal Through Enactive Cognition

**DOI:** 10.3389/fpsyg.2018.01129

**Published:** 2018-07-09

**Authors:** Kirsten A.-M. van den Bosch, David Welch, Tjeerd C. Andringa

**Affiliations:** ^1^SoundAppraisal Ltd., Groningen, Netherlands; ^2^School of Population Health, University of Auckland, Auckland, New Zealand; ^3^University College Groningen, University of Groningen, Groningen, Netherlands

**Keywords:** soundscapes, enactive cognition, evolutionary psychology, soundscape descriptors, soundscape indicators, audible safety, tranquility

## Abstract

We propose a framework based on evolutionary principles and the theory of enactive cognition (“being by doing”), that addresses the foundation of key results and central questions of soundscape research. We hypothesize that the two main descriptors (measures of how people perceive the acoustic environment) of soundscape appraisal (‘pleasantness’ and ‘eventfulness’), reflect evolutionarily old motivational and affective systems that promote survival through preferences for certain environments and avoidance of others. Survival is aimed at ending or avoiding existential threats and protecting viability in a deficient environment. On the other hand, flourishing occurs whenever survival is not an immediate concern and aims to improve the agent’s viability and by co-creating ever better conditions for existence. As such, survival is experienced as unpleasant, and deals with immediate problems to be ended or avoided, while flourishing is enjoyable, and therefore to be aimed for and maintained. Therefore, the simplest, safety-relevant meaning attributable to soundscapes (audible safety) should be key to understanding soundscape appraisal. To strengthen this, we show that the auditory nervous system is intimately connected to the parts of our brains associated with arousal and emotions. Furthermore, our theory demonstrates that ‘complexity’ and ‘affordance content’ of the perceived environment are important underlying soundscape indicators (measures used to predict the value of a soundscape descriptor). Consideration of these indicators allows the same soundscape to be viewed from a second perspective; one driven more by meaning attribution characteristics than merely emotional appraisal. The synthesis of both perspectives of the same person–environment interaction thus consolidates the affective, informational, and even the activity related perspectives on soundscape appraisal. Furthermore, we hypothesize that our current habitats are not well matched to our, evolutionarily old, auditory warning systems, and that we consequently have difficulty establishing audible safety. This leads to more negative and aroused moods and emotions, with stress-related symptoms as a result.

## Introduction

In this paper, we will use the conceptual framework of enactive cognition to address the foundation of key results and central questions of soundscape research. We will propose a theory based on evolutionary psychology, which underlies the identification of pleasantness and eventfulness as important soundscape descriptors.

Traditionally, research on noise (defined as unwanted sound) has focused on the relation between adverse effects and acoustic parameters such as level in decibels, dB(A). Cardiovascular diseases are one of the most studied adverse effects of noise exposure and include: hypertension, high blood pressure, ischaemic heart disease, and myocardial infarction (Ising and Kruppa, 2004; World Health Organization [WHO], 2011). These effects tend to be predicated (albeit implicitly) on the noise-stress hypothesis, under which noise is a non-specific stressor that activates the autonomic nervous system and endocrine system. This stress response elicits changes in stress hormones such as cortisol and (nor)epinephrine, affecting the individual’s metabolism, and increasing the risk for cardiovascular diseases. These effects seem to occur above noise levels around 65 dB(A) (Babisch, 2002; Ising and Kruppa, 2004). While these are valuable observations, they lack a suitable framework to explain the origins, effects, and workings of the noise-stress hypothesis. This theoretical basis is important, since it is becoming clear that auditory appraisal is greater than the sum of its decibels. In fact, the very definition of ‘noise’ as unwanted sound entails appraisal on the dimension of desirability that has no obvious relation to decibels.

The soundscape approach contributes to a growing body of research indicating that, for noise, it is not just objectively measurable signal properties, but the *meaning* attributed to it that has the most prominent effect on health (Ising and Kruppa, 2004). This coheres with phenomenological approaches to the relationship between individuals (or groups) and their environment (Von Uexküll, 1992/1934; Graumann, 2002) that focus on how meaning is constructed. From this perspective it is not surprising that merely one third of noise disturbance can be accounted for by acoustics alone (Guski, 2001). Research shows that sounds may be unpleasant due to the meaning attributed to them rather than their measurable energetic properties. Qualitatively unpleasant sounds (such as metal scraping on slate) can seem worse than electric shocks or neutral sounds presented at much higher levels (Neumann et al., 2008) and emotionally laden sounds elicit greater physiological responses (e.g., startle reflex, skin conductance) than neutral sounds of similar level (Bradley and Lang, 2000). Similarly, the mere reduction of noise levels does not necessarily lead to more positive appraisals of that environment (Adams et al., 2006; Dubois et al., 2006); on the contrary, it can even lead to (more) anxiety (Stockfelt, 1991).

By targeting the meaning of sound, soundscape research goes beyond the traditional focus on noise (Schulte-Fortkamp, 2002; Botteldooren et al., 2006; Cain et al., 2013) including both positive and negative effects on the perceiver. These effects could be attributed to very basic aspects of our perception. Auditory appraisal can even be seen as a basic requirement of life for humans as we have evolved, meaning it must be based on the environmental conditions for which our nervous systems evolved. The domain of enactive cognition (Varela et al., 1993; Thompson, 2007; Froese and Ziemke, 2009; Di Paolo et al., 2010) provides a conceptual framework to address questions related to the basic properties and role of soundscape, such as why pleasantness and eventfulness are crucial soundscape descriptors.

## Cognitive Foundations of the Soundscape Concept

The enactive approach of cognition sets out with the observation that life on Earth consists of individuals that remain alive because they *do* things to avoid premature death. This can be summarized as “being by doing” (Froese and Ziemke, 2009) and an entity that does this, within the domain of enactive cognition, is referred to as ‘an agent.’ This holds for all life: in single or multicellular living agents (organisms like humans and plants) this same basic function requirement of “being by doing” needs to be fulfilled. According to Barandiaran et al. (2009, p. 367) agency is *“an autonomous organization that adaptively regulates its coupling with its environment and contributes to sustaining itself as a consequence.”* This formal definition is a succinct formulation of a number of properties that living agents exhibit to remain alive and functioning. It entails the following:

1. An agent must not only be able to respond to the here and now, but be able to deal with its future demands as well. Agency is essentially anticipatory (Louie, 2010; Vernon, 2010).
2. Agents are continually adapting to the environment to ensure that they can sustain themselves. In practice, this entails that they satisfy their needs. Yet they are free to self-select their need satisfying behavior. This is called ‘needful freedom’ (Froese and Ziemke, 2009).
3. Unlike a rock or a hurricane, agents display (through their behavior) a measure of control over how they respond to and interact with the environment.

**FIGURE 1:**
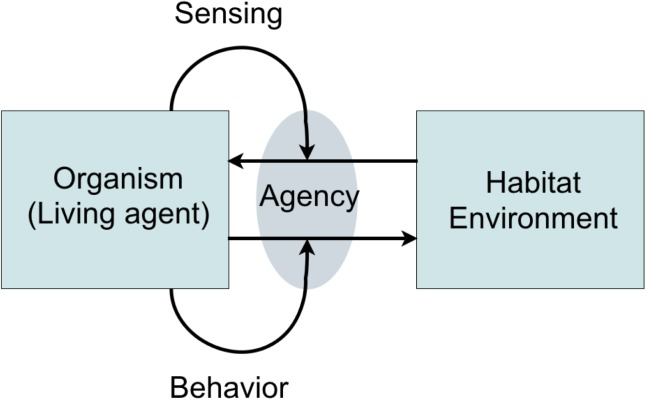
Agency. Agency is an organism’s ability to self-regulate its coupling to its habitat/environment through sensing and self-initiated activities (behavior). Adapted from Barandiaran et al. (2009).

The last property goes to the core of what it entails to be alive: agents act differently in different situations and the decision to act resides within the agent itself. While an inanimate object is only subject to external forces, an agent is a source of self-controlled modifications of its relation to the environment. In other words, it is agentic (**Figure 1**) (Barandiaran et al., 2009).

Agents sense the environment via specialized sensory systems which alter the internal state in response to the relevant observations of the environment (Egbert et al., 2010). Depending on a combination of what is sensed and the agent’s needs, behavior is selected. For example, this may entail the uptake of nutrients or a movement up or down some perceived gradient (Egbert et al., 2010). Evolution dictates that agents tend to optimize the functions of sensing and behavior so that outcomes are beneficial for survival. The combined process of sensing, behavior selection, and behavior enaction contributing to the agent’s continued existence and flourishing, is known as ‘cognition’ (Di Paolo and Thompson, 2014). From the perspective of cognition, the environment may be described as the combination of potential benefit or harm to the agent, and the investments the agent must make to respond. This constitutes the ‘affordances’ of the environment. The term affordance was first coined by Gibson (1979, p. 127), where he defined it as follows: *“The affordances of the environment are what it offers the animal, what it provides or furnishes, either for good or ill. […] It implies the complementarity of the animal and the environment.”*

Soundscape can be seen as the human sonic analog of this: for humans, the soundscape represents what the acoustic environment offers, provides, or furnishes the individual for good or ill. Definitions of soundscape refer accordingly to an “*acoustic environment as perceived or experienced and/or understood by a person or people, in context*” (International Organization for Standardization [ISO], 2014), or as Truax (1999, p. 126) described it, *“an environment of sound (sonic environment) with emphasis on the way it is perceived and understood by the individual, or by a society. It thus depends on the relationship between the individual and any such environment.”* An environment’s influence on agents depends upon the cognition it causes and the resulting meaning attribution in terms of affordances and the investments to realize them. It will be clear that the physical signals in the environment are a necessary precondition for meaning attribution. However, species specific innate processing capabilities, individual histories, social relations, and cultural knowledge usually dominate meaning attribution (Schafer, 1977). This implies that soundscape descriptors (measures of how people perceive the acoustic environment; Aletta et al., 2016) should reflect meaning attribution, as opposed to merely describing the physical properties of the sound itself (Cain et al., 2013). Such descriptors are addressed in the next section.

## Soundscape Descriptors: Pleasantness Versus Eventfulness

In parallel with the arguments based on enactive cognition, Bradley and Lang (2000) found that the principal variance in emotional meaning people give to sounds, can be explained by two (appetitive and defensive) motivational systems that underlie affective judgment; valence indicates which system is active, and arousal indicates the intensity of activation of these systems. Semantic descriptors of soundscapes appear to reflect a similar two-dimensional model for the underlying perceptual factors (Cain et al., 2013). Axelsson et al. (2010) named these ‘Pleasantness’ and ‘Eventfulness.’ Davies and Murphy (2012, p. 4) suggest that “*the weight of evidence in the literature is now sufficient for the first two dimensions of calmness/pleasantness and activity/eventfulness to be regarded as a ‘standard model’ for the perceptual dimensions of soundscapes.*” which is supported by a recent review on soundscape descriptors by Aletta et al. (2016).

**FIGURE 2:**
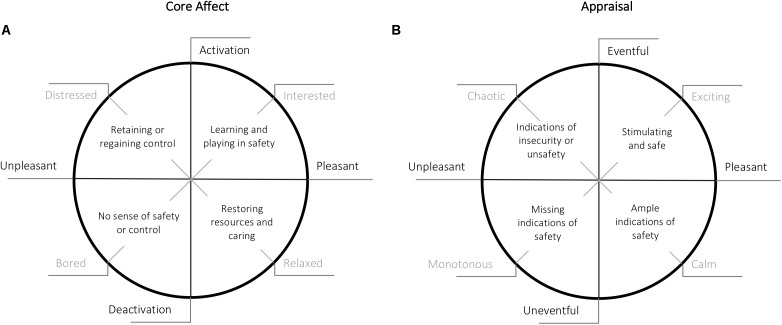
Core affect and appraisal. **(A)** Depicts core affect (Russell, 2003), while **(B)** depicts appraisal of the environment (Axelsson et al., 2010). Adapted from Andringa and Lanser (2013). The safety related remarks in the circle are addressed in Section “Audible Safety.”

It is important here to note the subtle yet substantial difference between descriptors of the affective quality of the environment (pleasantness and eventfulness) and descriptors of emotional responses to the environment (valence and arousal). The soundscape depends upon a combination of environmental influences on our senses (especially hearing), the process of ascribing meaning to the sensation of those influences (which may be termed perception), and the cognitive-emotional responses to the perception. By the definition we have used, the perception is the soundscape. Therefore, the soundscape depends on acoustical environmental cues and gives rise to psychological responses such as affective states, feelings, and cognitions. Furthermore, these psychological responses can in turn influence the perception of that environment (Schafer, 1977). The notion of this reciprocal relationship is supported by *in vivo* research on the way humans appraise their (current) environment and in what way that influences how they feel, plan, and act (Kuppens et al., 2012). From the perspective of an agent, the soundscape is the internal representation of the (mostly) acoustic environment, and the psychological responses to it are not necessarily clearly distinguishable from the soundscape itself. Thus, it can be difficult to separate these elements when considering the response of a person to an acoustic environment. To illustrate this distinction (and the similarities), **Figure 2** shows the two different elements or categories of descriptors.

Since the main descriptors of affective quality of the environment (Pleasantness and Eventfulness) closely resemble the concept of ‘core affect,’ this concept is used here to depict descriptors of emotional responses to the environment. Furthermore, both are often visualized as a circumplex model allowing for a side by side comparison. Core affect defines basic affective feelings that are always present and is an integral blend of the dimensions Pleasantness (valence) and Activation (arousal) (Russell, 2003). Core affect is a relation to the world as a whole and not a relation with something specific in that world. Like moods, it does not have (or need) the intentionality (directedness) of emotions and it is, unlike emotions, continually present to self-report. Following Kuppens et al. (2012), core affect reflects one part of the bidirectional relationship, the appraisal of the environment the other part.

Until now we have shown that pleasantness and eventfulness emerge as key soundscape descriptors from scientific literature. However, we argue that our theoretical basis allows to derive the same result from first principles. According to Andringa et al. (2015), agents exist in a superposition of two modes of being: (1) Survival (coping mode) and (2) Flourishing (co-creation mode). Survival is aimed at ending or avoiding threats to existence and protecting viability in a deficient environment. It is essentially problem-oriented, reactive, and self-centered. Flourishing occurs when survival is not an immediate concern and aims to improve the agent’s viability and to create ever better conditions for living (Fredrickson, 2001; Andringa et al., 2015). This corresponds to pervasive optimization, proactivity, and is environment-oriented, which has been connected to positive emotions and in particular to the broaden and build hypothesis (e.g., Fredrickson, 2001; Fredrickson and Branigan, 2005) using observations that positive emotions do not have a clear focus and broaden the scope of attention (Andringa et al., 2015).

We argue that the reactive survival (coping) mode is thus prevalent in low viability situations while the proactive flourishing (co-creation) mode is prevalent in high viability environments. As such, survival mode is experienced as unpleasant, and deals with immediate problems to be ended or avoided, while flourishing is enjoyable, and therefore to be aimed for and perpetuated (Andringa et al., 2015). These modes may be considered in terms of the two main descriptors of soundscapes: pleasantness and eventfulness. The absence of threats to survival and flourishing are perceived as pleasant states, whereas threats to survival or a lack of opportunities are unpleasant. Eventfulness is a dimension orthogonal to pleasantness and reflects the investment required to respond adequately to threats or opportunities. High investment environments lead to a high arousal level, while low investment environments allow low arousal.

To promote survival, our surroundings constantly influence our perception, cognition and emotions, even when we are not aware of it (Bitner, 1992). Therefore, as noted before, perception and the affective responses it elicits should not be considered separately: they are essentially intertwined (Kuppens et al., 2012). Perception impels our basic emotions (Izard, 2007) and our emotions serve to establish our position in our environment; they attract us toward places, situations, and people, where we can flourish, and they repel us from situations where survival is threatened or where it is difficult to flourish (Levenson, 1999). This push and pull, attraction and rejection, evaluation in terms of positive and negative, beautiful and ugly, good, and bad, is a central part of our lives and a cross-cultural phenomenon (Osgood, 1975). Wundt (1897) referred to this as affect, and he argued that these subjective experiences, or impressions of the world, in terms of good or bad (valence) are the most pervasive aspect of human perception. Similarly, Russell (2003) has described core affect as the heart of all affective experiences. The full range of highly positive and deeply negative emotional meanings that people attribute to sounds (Bradley and Lang, 1999) arises from an interaction between the listener, the listener’s attitude toward the sound source, the sound source itself, and other context (Tajadura-Jiménez, 2008). These insights describe a deep and essential mutual influencing of the state of the individual and the appraised environment, which implements the notion of agency as defined in **Figure 1**. In fact, the variety of relations between individual and environment as described in the previous paragraph and the key results of soundscape research are all perspectives on agency.

## Audible Safety

The abovementioned findings suggest that environments are processed based on characteristics beneficial for survival and below we outline why this assumption holds for auditory perception. Hearing is a universal sense (Horowitz, 2012) since no animal species has evolved without an acute sense of hearing (unlike vision), and it has an evolutionary history of several hundreds of millions of years (much older than vision; Hester, 2005). Considering that, the auditory system’s most important function and original *raison d’être* (with respect to other senses) would then be to estimate danger and safety (Juslin and Västfjäll, 2008; Andringa and Lanser, 2013; Andringa and van den Bosch, 2013). Sound is perceived omnidirectionally, independently of lighting, physical obstructions, or wakefulness, and has strong attention-capturing power (Fritz et al., 2007). Furthermore, humans have an attentional bias for sounds outside the visual field (Tajadura-Jiménez et al., 2010b), with such sounds eliciting more arousal and larger physiological responses (Tajadura-Jiménez et al., 2010a), and humans have faster reaction times to auditory than to visual stimuli (Jasìkowski et al., 1990). These findings, together with our proposed evolutionary perspective, imply that audible safety might be the central element in the appraisal of our acoustic environments.

**FIGURE 3:**
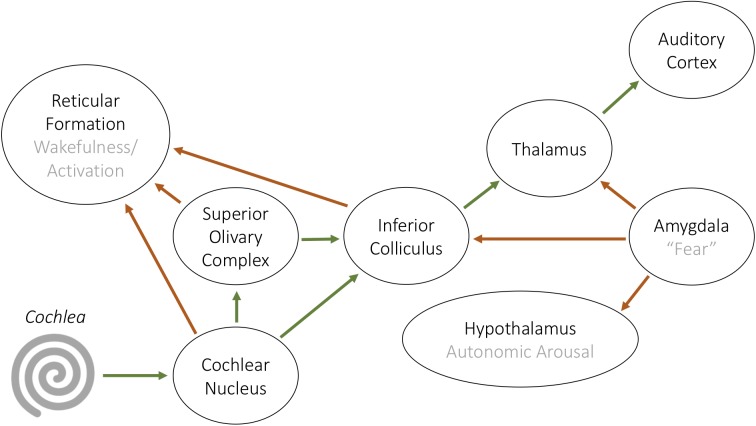
Schematic diagram of the classical auditory pathway (green arrows) and its associations with structures related to psychophysiological responses to soundscapes (red arrows).

In line with this, the auditory nervous system is intimately connected to the parts of our brains associated with arousal and emotions (**Figure 3**). The reticular formation is a distributed network of nuclei in the brainstem and has control over arousal and many aspects of brain activity (Brown et al., 2012). Inputs from the most peripheral nuclei in the auditory pathway, the cochlear nucleus and superior olivary complex, innervate the reticular system’s caudal pontine nucleus (Koch and Schnitzler, 1997). This operates in parallel and interactively with the classical auditory pathway to influence our experience of sound, and is also involved in other sensory systems, initiation and control of motor activity, autonomic arousal, sleep and wakefulness, and emotions (Siegel and Sapru, 2011). The system provides one mechanism for the emotional impact of sound, and it may influence the physiological and thus the emotional response to stimuli that have salience for survival and are treated as important.

The midbrain mediates freezing and flight in the face of alarming sounds as well as containing the limbic system, where emotional responses are mediated (Spreng, 2000; Ising and Kruppa, 2004; Kraus and Canlon, 2012). The auditory system projects information via the inferior colliculus and the medial geniculate nucleus of the thalamus, to the auditory cortex. The inferior colliculus, with involvement from the auditory cortex, directs flight from sudden, loud sounds via the superior colliculus and the periaqueductal gray (Xiong et al., 2015). The medial geniculate nucleus also projects to the amygdala, where emotional valence is attributed to sound (Kraus and Canlon, 2012). Furthermore, the amygdala itself has projections back into the auditory system (the inferior colliculus), implying that there may be modulation of auditory signals depending upon the emotional/meaningful/safety-related content in them (Marsh et al., 2002).

These observations allow us to propose that the brain constantly responds not only to the acoustic aspects of sounds but also to deeply programmed affectual, arousing, and attention-grabbing aspects of sounds. These two aspects of the response to sound occur in parallel and with feedback. From the perspective of the (human) agent, the two aspects of the percept (perception of the acoustics and meaning attribution) are inextricable. This allows to design for desired forms of audible comfort by separating the attention-grabbing foreground from a background that continually provides us with a sense of place. If this is a sense of a *safe* place – because the midbrain is able to estimate ample indicators of safety – the listener is allowed full freedom and control to self-regulate mind-states according to needs (Andringa and Lanser, 2013; Andringa and van den Bosch, 2013).

In **Figure 2**, the relation between indicators of (audible) safety, affective appraisal of soundscapes, and core affect is illustrated. Here, it can be seen that pleasantly appraised environments co-occur with a pleasant inner affective state, proactive behavior, and (at least) ample indicators or safety. In the absence of indications of safety or presence of indications of unsafety, an environment is perceived as unpleasant, on which the agent will reply with reactive behavior, to avoid or end an unpleasant inner affective state (core affect). More specifically: a calm environment affords ample indications of safety that allow us to restore our resources and to care for self and environment; a lively environment is a stimulating and safe place that allows us to learn and play; a boring environment misses indications of safety, which does not afford a sense of safety or control; and a chaotic environment contains clear indications of insecurity or unsafety and forces to retain or regain control.

To summarize, we hypothesize that our appraisal of soundscapes is based on old survival-driven strategies, and we propose that the first (subconscious) decision made in the processing of auditory information is an assignment of safety by subcortical processes. Only in the case of a predominance of positive indicators of safety, will listeners have full freedom and control over mind-states. If not, part of the cognitive resources will be involved in general vigilance or directed attention to potential threats (Andringa and Lanser, 2013).

## Soundscape Indicators: Complexity Versus Affordances

By accepting pleasantness and eventfulness as the main affective descriptors of soundscape appraisal, “*the hunt*” for the underlying indicators has begun; these are defined as “*measures used to predict the value of a soundscape descriptor*” (Aletta et al., 2016). Our evolutionary perspective and the concept of audible safety provide clues about them.

We propose that the second set of soundscape descriptors, calmness and excitement, as proposed by Axelsson et al. (2010) (or calmness and vibrancy as found by Cain et al., 2013), actually reflect the indicators ‘Complexity’ and ‘Affordance Content,’ respectively (Andringa and van den Bosch, 2013). This interpretation allows for an explanation that draws on our proposed evolutionary theory, while maintaining the essential two-dimensional space. Here, affordances are the threats and opportunities in an environment (Gibson, 1979) and indicate the extent to which the environment offers options for self-selected behavior and self-regulation (Andringa et al., 2015). The complexity of an environment refers to the number of competing auditory streams (Bregman, 1994), and thus how difficult it is to process the available affordance content (Axelsson, 2011) and to choose situationally appropriate behavior. The larger the search space and the smaller the set of beneficial options, the more complex and demanding the decision-making process that we refer to as ‘*meaning attribution.’* The observation that the appraisal of the environment in part depends on the degree of perceived control (Russell, 2003) is illustrative of the influence complexity has on perception.

The new dimensional structure of indicators can be seen to have parallels in the prospect refuge theory (Appleton, 1975). Natural environments may be (visually) analyzed based on structural aspects such as, depth, threats, and opportunities (e.g., navigability, concealment), which elicit affective responses mediating adaptive behavior, and as such promote survival (Appleton, 1975; Ulrich, 1983; Greene and Oliva, 2009). Although the prospect refuge hypothesis was originally formulated for landscapes, soundscapes help us just as much in characterizing different environments (Pheasant et al., 2010) and determining survival relevant meaning. Schafer (1977) definitions of high-fi and low-fi soundscapes was already suggestive of this function. A hi-fi soundscape has little overlap of the foreground sounds, and the sounds from the wider surroundings. This allows for a distant sonic horizon and a high signal-to-noise, or foreground-to-background, ratio. Alternatively, low-fi soundscapes are associated with an industrial, mechanized world and have sonic horizons that are much closer (Schafer, 1977). As such, a high-fi (often natural) soundscape is favorable for survival purposes since it makes the signal easier to process (Andringa, 2002), which reduces the processing complexity of its analysis.

**FIGURE 4:**
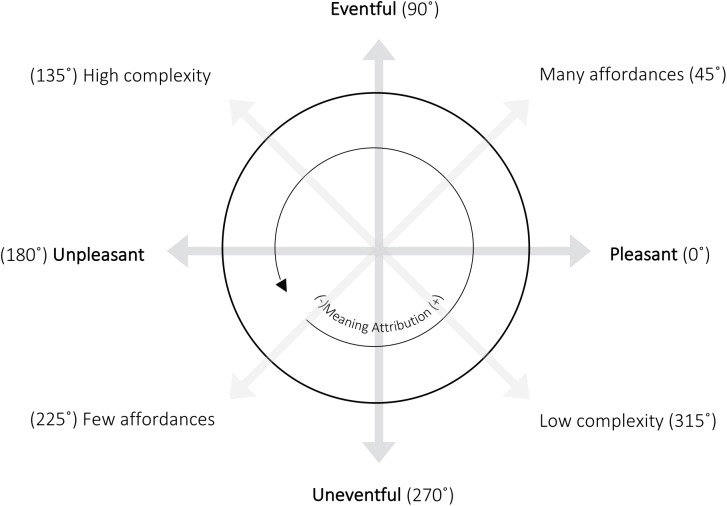
Two-dimensional model of soundscapes with the main descriptors pleasantness and eventfulness, the underlying indicators complexity and affordances, and the relation to meaning attribution. The indicated degrees are used as a guideline for further explanation in **Table 1**.

To illustrate the above-mentioned findings, **Figure 4** integrates the main descriptors of soundscapes with the proposed underlying indicators and the relation to meaning attribution in terms of enactive cognition’s central notion of “being by doing.” The horizontal axis represents the soundscape descriptor pleasantness (a measure of ‘being’). The vertical axis represents the soundscape descriptor eventfulness (associated with ‘doing’). The diagonal axes represent the indicators affordance content (need satisfaction benefits) and complexity (of action selection). Meaning attribution is a function of the indicators affordance content and complexity, as described by Axelsson (2011), and as such could actually be viewed as a (compound) soundscape indicator in itself, influencing the perceived quality of soundscapes.

**Table 1** elaborates on eight possible positions in this two-dimensional model and interprets them in terms of the meaning attribution. It uses the degrees as depicted in **Figure 2** and starts from 225°: what Eckblad (1981) referred to as the ‘empty sector.’ We do that because this sector is interpreted slightly differently when approached from the 180° direction, where it corresponds to an inability to attribute meaning, than from the 270° direction, where it corresponds to the absence of useful affordances (hysteresis). Both interpretations lead to the lowest rate of useful meaning to be estimated from the signal, in terms of satisfying agentic needs.

**Table 1:** Soundscape indicator-based descriptions of the two-dimensional model of soundscape appraisal as depicted in **Figure 4**.

Angle	Description in terms of affordances (threats and opportunities), complexity (to analyze the sonic environment and select behavior), indicators of audible safety, viability, and investment	Meaning attribution interpretation
225°	An absence of useful perceived affordances leads to a minimal search space for situationally appropriate behavior. Minimal agency and problematic viability. Medium complexity environment due to unsuccessful estimation of audible safety, for example because indicators of safety might be masked by other sounds.	Minimal meaning attribution in combination with unsuccessful safety estimation
270°	Few, neither safe nor dangerous perceived affordances, deep relaxation associated with the absence of an urge to invest in interaction. Low complexity environment. Audible safety indicators either somewhat present or indicators of unsafety absent yet in principle easily audible because they are not masked by other sounds (silence).	Very little meaning to be attributed
315°	Normal level perceived affordances with abundant indicators of safety and an (audible) absence of threats, which makes it very easy to select behavior. Minimal investment required in environmental interaction. Minimally complex environment. Allowing for full freedom and control to self-regulate mind-states according to needs and desires.	Meaning attribution very easy
0°	Many perceived opportunities, ample indications of safety, maximally beneficial, highest viability, easy to select behaviors, yet requiring investment in environmental interaction. Low complexity environment.	Meaning attribution easy
45°	Maximum level of perceived opportunities, ample indications of safety. This leads to a large search space in which a beneficial choice is neither crucial nor harmful. However, exploration of the rich affordances space requires a fairly high investment. Medium complexity environment.	Rich meaning attribution
90°	High perceived affordances, but now not necessarily with safe outcomes and/or weaker indications of safety. Still large space for behavior selection, but with a smaller set of beneficial outcomes, which makes it difficult to select beneficial behaviors and avoid harmful outcomes. Requires maximal investment in environmental interaction. High complexity environment.	Meaning attribution challenging
135°	Focus on potentially or actually *unsafe* perceived affordances (threats - indications of danger). Ignoring parts of the sonic environment, without sufficient indicators that these parts are safety irrelevant. Behavior selection is crucial to select the few options that are not harmful (and perhaps find the even fewer that are beneficial). Environmental interaction focused on these options. Maximally high complexity environment requiring a high investment.	Information overload, focus on subset, meaning attribution incomplete
180°	Low level of perceived affordances. Threats and indications of danger are dominant and prevent an adequate analysis of potentially relevant content. Only few behavioral options are not dangerous. Behavioral choices become limited to the few that are not beneficial or even harmful. High complexity environment in which analysis efforts do not pay off, leading to a sense of agentic inadequacy.	Information overload and processing inability, attributed meaning attribution crucial but not satisfactory
225°	Minimal level of perceived affordances all with minimal options, the behavioral selection search space may not include solutions so that the individual feels trapped and is subject to environmental influences. This is a medium complexity environment because the event rate to attend is not high, yet unable to address unfulfilled needs. This leads to a minimal sense of agency in an environment in which investment opportunities are low.	Meaning attribution unable to satisfy needs despite efforts

Note that the agent should always remain responsive to possible developments in the environment. This entails that it cannot spend more resources than it can muster before the situation changes. Perception is always under time pressure and hence processing resources are finite. Highly complex environments may change before meaning can be attributed reliably, which puts the agent under time-pressure to decide on the basis of insufficient information. If this is the case, the agent is unable to reliably determine audible safety and/or other relevant affordances. Alternatively, in an environment devoid of affordances, the perceiver is equally unable to determine audible safety and other meaning, however, much it searches for these. Hence, from 225° we go anti-clockwise via environments that become progressively more complex to environments after 90° that are so complex that they cannot be processed in full, and finish back at 225° in environments of which only superficial real-time meaning can be attributed.

## A Practical Implication: The Need for Tranquility

Our evolutionary perspective on soundscapes allows the formulation of some practical implications which should be considered in the design of soundscapes. For example, we propose that environments which are dominated by mechanical sounds, will effectively mask natural sounds that are preferred by our auditory sensory system to estimate audible safety. This is supported by findings indicating that mechanical sounds decrease perceived tranquility, and natural sounds enhance it (Pheasant et al., 2010). Similarly, findings by Darner (1966) demonstrated that mechanical sounds elicited unpleasant and alert feelings (as opposed to the sound of birds), and more recently Buxton et al. (2012) found that electronic sounds are more arousing than other sounds of similar loudness.

Our urbanized societies have become more mechanical, less harmonious, less predictable and controllable, leading to more negative appraisals of the (urban) soundscapes we live in (Davies et al., 2009). This results in a universal need for quietness (Pheasant et al., 2010; Booi and van den Berg, 2012), which can be explained by the Attention Restoration Theory of Kaplan (1995). The Attention Restoration Theory states that prolonged periods of (subconscious) directed attention lead to attentional fatigue, which needs to be recovered in restorative environments. This gains support from findings that restorative environments offering relief from sustained directed attention (associated with high complexity processing) are known to reduce stress and increase well-being (Hartig et al., 1997). For restoration, we need an alternate mode of attention, one that benefits recovery: fascination. It is proposed that natural environments are ideally suited for fascination because they are tranquil, leave a harmonic impression, and are rich, yet do not demand directed attention (Kaplan, 1995; Booi and van den Berg, 2012; Payne, 2013). We suggest this is due to the high redundancy of easy to process indications of audible safety in most natural environments. Therefore, our environments should offer more diversity through better access to green and natural spaces, especially in busy cities, so that people have access to tranquil (and audibly safe) soundscapes where they can recover from our cacophonous habitats (Booi and van den Berg, 2012).

## Future Directions

Pleasantness and eventfulness, and their indicators affordances and complexity, are predicted by the evolutionary cognitive theory we have described above. However, does this two-dimensional model truly and fully describe the soundscape, or might there be other important dimensions that could be predicted by the framework? It should account for all descriptors that would contribute to evolution, which includes dimensions of perceived affective quality such as pleasantness and eventfulness, but also descriptors from other categories. One candidate is ‘appropriateness,’ which has been mentioned in research Aletta et al. (2016). Soundscape appraisal is highly variable across intended activities (Nielbo et al., 2013; Steele et al., 2015), and expectations and appropriateness seem to play a significant role in the evaluation of soundscapes Aletta et al. (2016). Any activity or state draws on information schemes (Eckblad, 1981) and the encountered situation is matched against the existing cognitive schemes of information. A match between the scheme and the real-world situation leads to pleasant affective responses to stimuli based on affirmation and security, whereas a mismatch (inappropriateness) leads to negative affective responses, confusion and insecurity. Appropriateness thus makes for very personal assessments of environments which are only suitable for specific situations or places (Brown et al., 2011). In the context of a soundscape, appropriateness would reflect the extent to which aspects of the acoustic environment matched the scheme in the mind of the listener. Sound elements which did not match (for example a car motor in a wilderness) would be perceived as inappropriate. In terms of evolutionary theory, the capacity to detect inappropriate elements would indeed be crucial for survival, and thus such a soundscape dimension may be expected to exist.

There are many other possible factors which appear to play a role in our appraisal of soundscapes. For example, a sense of pace or the passage of time, feelings of spirituality associated with the sonic environment, and an awareness of spaciousness, have all been identified using an essentially atheoretical approach to observing the soundscape (Welch et al., unpublished). Other research and theoretical work relating to the appreciation of loud music represents an understanding of an (artificial) soundscape, and concepts such as feelings of power or personal strength, and an experience of being transported to other worlds or imaginary realities have been reported (Blesser, 2007; Welch and Fremaux, 2017a,b). These qualities of the soundscape do not seem to be captured by the pleasantness/eventfulness dimensions and nor are they yet incorporated into the theoretical stance we have proposed here. Widening our understanding of the soundscape may be possible on both a practical and a theoretical level. On a practical level, we may gradually increase the dimensionality, or else learn how to apply different dimensionalities according to the physical/perceptual environment to allow these qualities to be incorporated.

On a theoretical level, we may be able to apply the evolutionary/cognitive approach we have proposed here to some of these other qualities. Alternatively, a compound theory which also draws upon other positions than the evolutionary may be necessary. Application of enactive cognition theory to explain the (apparently) more fundamental aspects of the soundscape (e.g., time) seems feasible. For example, any agent must operate with time constraints and we have therefore evolved to be able to do this. Our awareness of time passing would reflect an ability which evolved to allow us to make judgments about probabilities of survival and flourishing in future: this then may represent another theoretical dimension of our emotional appraisal, and may therefore provide a theoretical basis for future explorations of the soundscape. More careful thinking will be necessary to consider these possibilities, but the development for a strong theoretical basis to help drive and interpret soundscape research is crucial.

## Conclusion

Based on an evolutionary theory in which agents are motivated to seek pleasant and avoid unpleasant environments with the intention to flourish, we have bolstered the theoretical underpinning to a two-dimensional model of soundscape appraisal. We have shown that, according to our theory, (1) the main soundscape descriptors pleasantness and eventfulness arise by necessity and (2) that affordance content and complexity of behavior selection are underlying indicators of these soundscape descriptors. Our theoretical basis comprises the defining properties of life and cognition (as formulated in the domain of enactive cognition), which lead to the formulation of constraints and opportunities afforded by living in a sonic world that underpin the science of soundscapes. Since our auditory sensory system can be regarded as an important warning system, and people appraise their soundscapes based on the level of safety they attribute to them, we propose that the simplest, safety-relevant meaning attributable to soundscapes is of central importance in understanding human perception.

Our approach allows the same soundscape to be formulated from a second perspective; one driven more by meaning attribution characteristics than merely emotional appraisal. The synthesis of the proposed indicators and the most common descriptors of soundscapes provides both perspectives of the same person–environment interaction, which consolidates the affective, informational, and the activity related perspectives on soundscape appraisal. Furthermore, we hypothesize that our current habitats are not well matched to our, evolutionarily old, auditory warning systems, and that we consequently have difficulty establishing audible safety. This leads to more negative and aroused moods and emotions, with stress-related symptoms as a result. A return to more natural sounding environments, or the design of non-natural environments with less threatening and less impoverished qualities, is the best guarantee for providing environments that are optimized for human inhabitants.

## Author Contributions

All authors contributed equally to this manuscript and developed the theory interactively. KvdB initiated the collaboration, wrote the first draft, and managed the writing process. DW provided most of the neurological information. TA added the perspective from enactive cognition.

## Conflict of Interest Statement

The authors declare that the research was conducted in the absence of any commercial or financial relationships that could be construed as a potential conflict of interest.
